# A Nation-Wide Multicenter 10-Year (1999-2008) Retrospective Clinical Study of Endocrine Therapy for Chinese Females with Breast Cancer

**DOI:** 10.1371/journal.pone.0100159

**Published:** 2014-07-18

**Authors:** Can Zhou, Jian jun He, Jing Li, Jin hu Fan, Bin Zhang, Hong jian Yang, Xiao ming Xie, Zhong hua Tang, Hui Li, Jia yuan Li, Shu lian Wang, You lin Qiao, Rong Huang, Pin Zhang

**Affiliations:** 1 Department of Oncology Surgery, First Affiliated Hospital, School of Medicine of Xi'an Jiaotong University, Xi'an, Shaanxi Province, China; 2 Department of Cancer Epidemiology, Cancer Institute & Hospital, Chinese Academy of Medical Sciences & Peking Union Medical College, Beijing, China; 3 Department of Breast Surgery, Liaoning Cancer Hospital, Shen yang, Liaoning Province, China; 4 Department of Breast Surgery, Zhejiang Cancer Hospital, Hangzhou, Zhejiang Province, China; 5 Department of Breast Oncology, Sun Yat-Sen University Cancer Center, Guangzhou, Guangdong Province, China; 6 Department of Breast-thyroid Surgery, Xiangya Sencod Hospital, Central South University, Changsha, Hunan Province, China; 7 Department of Breast Surgery, the Second People's Hospital of Sichuan Province, Chengdu, Sichuan Province, China; 8 Department of Epidemiology, West China School of Public Health, Sichuan University, Chengdu, Sichuan Province, China; 9 Department of Radiotherapy, Cancer Institute & Hospital, Chinese Academy of Medical Sciences & Peking Union Medical College, Beijing, China; 10 Department of Medical Oncology, Cancer Institute & Hospital, Chinese Academy of Medical Sciences & Peking Union Medical College, Beijing, China; University of North Carolina School of Medicine, United States of America

## Abstract

Endocrine therapy (ET) is one of the main systemic treatments for patients with breast cancer. To our knowledge, few studies have addressed the performance of ET or relevant influencing factors in cancer treatment in China. By retrospectively analyzing the clinicopathological data on breast cancer collected from representative hospitals of 7 traditional areas in China in one random month from each year between year 1999 and 2008, we found that: 1) The rate of the use of hormone receptor (HR) testing was 83.8% (3529/4211), with the estrogen receptor-positive (ER+) rate and/or the progesterone receptor-positive (PR+) rate being 67.9% (2395/3529), and the ER-PR rate being 32.1% (1134/3529). 2) Of the 1599 patients who had received ET, 999 patients (58.3%) were premenopausal while 600 (41.7%) were postmenopausal; 1598 patients received adjuvant hormonal therapy (AHT), whereas only 1 patient received palliative therapy. The medications mainly administered to patients were anti-estrogen agents (80.3% [1283/1598]), followed by AIs (15.5% [248/1598]). Of the 1598 patients receiving AHT, 1416 patients (88.6%) were positive for ER and/or PR, while 75 (4.7%) were negative for both and 108 patients (6.7%) had unknown HR status. The ratio of the use of endocrine therapy for breast cancer patients with ER+ and/or PR+ status was 60.0% (1416/2395). 3) Results from the logistic regression analysis revealed that geography, occupations, and history of chemotherapy and surgery were dependent factors affecting the application of ET in breast cancer treatment in China (P<0.001). In conclusion, the use of ET on Chinese women with breast cancer is increasingly and gradually accounted into the standardized process. Economic status, occupations, and history of chemotherapy and surgery were key factors affecting the application of ET. People residing in developed areas, engaging in mental labour, having history of chemotherapy and surgery are susceptible to accept ET.

## Introduction

The application of endocrine therapy (ET) can be traced back to year 1896, when the English scholar Beatson performed oophorectomies for treatment of pre-menopausal advanced breast cancer[6]. Since then, ET has become one of the main systemic treatments for patients with breast cancer[1]. Surgery for removal of endocrine glands, such as adrenalectomy[3]–[4], oophorectomy[4], or hypophysectomy[3]–[5], and administration of medications such as androgens[6]–[7], estrogen[5], anti-estrogens (tamoxifen) [8]–[11], or aromatase inhibitors (AIs) [12]–[22] have been used as part of a comprehensive breast cancer treatment plan.

Recently, hormone receptor tests have been widely used as part of pathologic examinations for breast cancer, including estrogen receptor (ER) and progesterone receptor (PR) tests. The expressions of ER and PR are important indicators for guiding breast cancer ET decisions and determining ET strategies [23]–[25]. The result of ER testing is an indicator for deciding whether the breast cancer is hormone-dependent (ER-positive) and hormone-independent (ER-negative)) [23]–[24].

An increasing number of clinical studies have revealed that ET has crucial implications for early-stage hormone-dependent (ER+ and/or PR +) breast cancer and recurrent and metastatic breast cancer [8]–[11] [14]–[22]. Indeed, ET can significantly reduce the risk of recurrence and metastasis [8]–[11], [14]–[22], thus increasing the overall survival rate of breast cancer patients[17], [24]. Moreover, ET has advantages over surgery, radiotherapy or chemotherapy in that it is easy to administer, causes no damage to normal tissues, and has fewer side effects but long-lasting efficacy[24], [26]. Therefore, ET has received increasing attention and gradually become part of the standardized treatment for breast cancer. Of note, the ET association studies in China are restricted mostly to several regions. And only a few studies have addressed the performance of ET and the factors that influence ET [27].

In this study, we retrospectively analyzed the clinicopathological data of 4211 breast cancer female patients collected from representative hospitals of 7 traditional areas in China in one random month from each year between 1999 and 2008. The data collected included hormone receptor tests, ET status and possible influencing factors. This study was aimed to improve the understanding of the current status of ET and the factors that influence ET in breast cancer treatment throughout China and to enhance our awareness of ET for patients with breast cancer.

## Methods

### Study design and data collection

We conducted a nationwide, multi-center retrospective clinical epidemiologic study of female breast cancer over a 10-year interval (1999–2008) in China. The study protocols were approved by the Cancer Foundation of China's Institutional Review Board.

The hospital selection and case sampling methods have been previously described in detail [28]. In brief, one tertiary hospital was selected in each of seven geographic regions of China (north, northeast, northwest, central, eastern, southern, and southwest). The regions cover most areas of the country and represent different levels of breast cancer burden. We selected the leading regional public cancer hospitals and referral centers which provide pathologic analysis, diagnosis, surgery, radiotherapy, medical oncology, and routine follow-up care for breast cancer patients. Another criterion was that that all patients were native residents in the study regions). One month was randomly selected from each year between 1999 and 2008 for collection of inpatient data in each hospital. January and February were excluded from the random selection to decrease any confounding effects of the largest annual holiday (the Spring Festival) in China. At least 50 pathologically-diagnosed inpatient cases selected from that month each year were enrolled for the review. If <50 qualifying cases were available in a selected month, the cases from the two months immediately before and after the selected month were included.

### Patient Selection and Pathologic Diagnostic Criteria

The patient selection and pathologic diagnostic criteria have been previously described in detail[28], [29]. All the patients enrolled in the current study were required to meet the following three inclusion criteria[28], [29]:i) primary breast cancer was confirmed by pathology;ii) inpatient admission date were within the selected month in the study hospital; and iii) the patients had received or were receiving treatment (surgery, medical oncology, and/or radiotherapy) for breast cancer. The cases were classified into histologic subtypes according to the 1981 and 2003 WHO histologic classification criteria[28],[29].Staging of breast cancer was performed according to the 1997 AJCC TNM staging system[28]. A semi-quantitative method was used to describe the results of ER and PR testing. The results were recorded in the form of the Harvey's report [30].

### Data Collection and Quality Control

As described in detail previously [28], [29], the following data were systematically collected for all enrolled patients via medical chart review:i) general information, including date of diagnosis, date of hospital admission, admission diagnosis; ii) demographic characteristics at the time of diagnosis/admission, including age, occupation, education, and marital status;iii) breast cancer risk factors, such as age of menarche, age of menopause, family history of breast cancer;iv) results of clinical breast examination (CBE);v) results of diagnostic imaging, including mammography and ultrasonography;vi) use of currently available surgical approaches, radiotherapy,chemotherapy and molecular targeted therapy for breast cancer; and vii) pathologic characteristics, including post-surgery pathology, ER and PR expressions, and human epidermal growth factor receptor (HER)-2 expression.

All of the above information was extracted from medical charts to designed case report forms (CRF) by local clerks after training. Then, two data input clerks from each hospital were recruited to independently double-enter data from the paper to a computer-based database (FoxPro). All of the completed double-entry databases were sent to Chinese Academy of Medical Sciences (CICAMS) for validation by running EpiData. Inconsistencies between the two databases revealed by CICAMS were reported to the local clerks for adjudication until the databases were consistent. As a final inspection, one of the databases was chosen to undergo a final consistency check. Logical errors (e.g., a patient who had intra-operative frozen section diagnosis, but no surgery) were reported back to the local hospital, and the local collaborators reviewed the original medical chart again. After checking with the original medical record, the local staff sent the revised database back to CICAMS for final analysis. During the consistency check, 5% of the medical charts were randomly selected based on the study ID and sent to CICAMS for quality control review.

### Data Analysis

Descriptive statistics were used to analyze the overall status of hormone receptor testing and ET. Cochran-Armitage time-trend analysis was used to determine the ratio of the use of hormone receptor testing or ET in each of the 10 years. Logistic regression analysis was used to determine the factors which influenced the use of ET. For all tests, two-sided p values <0.05 were considered statistically significant. All analyses were performed using SPSS software (version 13.0).

## Results

### Analysis of HR Testing

Among the 4211 patients with breast cancer, 3529 cases (83.8%) had estrogen receptor (ER) and progestin receptor (PR) tests. The median age of the patients was 48 years old (range, 21∼86 years), and the mean age was 48.8 years (±10.37). Overall, two testing techniques were used. 99.97% (3528/3529) cases were examined by immunohistochemistry while 0.03% (1/3529) by enzyme-linked immunosorbent assay (ELISA).

### Timing and Regional Analysis of HR Testing

**Figure 1:**
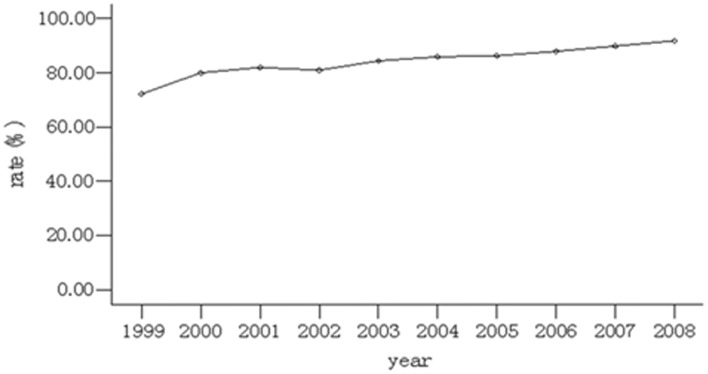
The trend of the use of hormone receptor testing during 1999 and 2008.

The rate of HR testing gradually increased from72.2% (291/403) in 1999 to 91.8% (456/497) in 2008, with statistically significant difference between the cases selected for each year (χ2 = 145.91,P<0.001; Figure 1). In the regional analysis, the rate of HR testing was the highest 98.0% (535/546) in the northeast area, and the lowest 58.3% (282/483) in the northwest area, with significant difference between the cases selected from each region during 1999 and 2008 (χ2 = 44.45,P<0.001; as shown in Table 1 and Figure 1).

**Table 1:** Hormone receptor testing status in different geographic areas during 1999 and 2008.

	Northern		Northeast		Central		South		Southeast		Northwest		Southwest		Total	
	n(%)	N	n(%)	N	n(%)	N	n(%)	N	n(%)	N	n(%)	N	n(%)	N	n(%)	N
1999	47(94.0)	50	52(100.0)	52	29(59.2)	49	35(58.3)	60	74(77.9)	95	7(14.9)	47	47(94.0)	50	291(72.2)	403
2000	44(88.0)	50	48(100.0)	48	23(46.0)	50	38(69.1)	55	39(75.0)	52	8(17.8)	45	44(88.0)	50	244(80.0)	305
2001	51(98.1)	52	46(100.0)	46	35(70.0)	50	53(91.4)	58	67(94.5)	74	14(29.8)	47	43(86.0)	50	309(82.0)	377
2002	38(92.7)	41	46(100.0)	46	32(62.8)	51	53(91.4)	58	44(86.3)	51	22(50.0)	44	41(82.0)	50	276(81.0)	341
2003	50(100.0)	50	49(100.0)	49	37(71.2)	52	54(91.5)	59	65(83.3)	78	31(58.5)	53	43(87.8)	49	329(84.4)	390
2004	47(94.0)	50	51(100.0)	51	56(87.5)	64	44(83.0)	53	79(82.3)	96	43(81.1)	53	38(76.0)	50	358(85.9)	417
2005	57(95.0)	60	52(98.1)	55	43(74.1)	58	51(86.4)	59	63(84.0)	75	39(79.6)	49	45(90.0)	50	350(86.2)	406
2006	86(98.9)	87	56(93.3)	60	55(80.9)	68	51(96.2)	53	77(81.1)	95	38(77.6)	49	43(86.0)	50	406(87.9)	462
2007	108(98.2)	110	66(100.0)	66	73(88.0)	83	67(90.5)	74	108(80.6)	134	40(78.4)	51	48(96.0)	50	510(89.8)	568
2008	88(96.7)	91	69(94.5)	73	79(97.5)	81	65(86.7)	75	69(84.2)	82	40(88.9)	45	46(92.0)	50	456(91.8)	497
Total	616(96.0)	641	535(98.0)	546	462(76.2)	606	511(84.6)	604	685(82.3)	832	282(58.3)	483	438(87.8)	499	3529(83.8)	4211

χ2area = 44.45,P<0.001.

### Analysis of HR composition

Of the 3529 patients undergoing HR testing, a total of 2395 (67.9%) cases were hormone receptor positive (ER and/or PR positive; ER+ and/or PR+) breast cancer, with a median age of 48 years (range, 21∼86 years) and a mean age of 48.72 (±10.40) years.

Among the 2395 patients with hormone receptor-positive breast cancer, 1691 (47.9%) cases were positive for ER and PR (ER+PR+), 337 (9.6%) cases were positive for ER but negative for PR (ER+PR-), 367 (10.4%) cases were negative for ER but positive for PR positive (ER-PR+), and the remaining 1134 cases (32.1%) were negative for both (ER-PR-).When it comes to regional factors, we found that the highest proportion of patients with ER+PR+ resided in north China (61.2% [373/610]), the highest proportion of patients with ER+PR- resided in southwest China (11.8% [51/431]), the highest proportion of patients with ER-PR+ resided in northeast China (15.2% [108/711]), and the highest proportion of patients with ER-PR- resided in middle China (38.1% [203/533]). The differences between the groups were statistically significant (χ2 = 794.228,P<0.001; Table 2).

**Table 2:** Status of HR in patients from different geographic areas.

	Northern		Northeast		Central		South		Southeast		Northwest		Southwest		Total	
HR type	n	%	n	%	n	%	n	%	n	%	n	%	n	%	n	%
ER+PR+	373	61.2	318	44.7	244	45.8	241	47.7	221	47.9	109	39.2	185	42.9	1691	47.9
ER+PR-	44	7.2	79	11.1	54	10.1	37	7.3	41	8.9	31	11.2	51	11.8	337	9.6
ER-PR+	47	7.7	108	15.2	32	6.0	75	14.9	34	7.4	37	13.3	34	7.9	367	10.4
ER-PR-	146	23.9	206	29.0	203	38.1	152	30.1	165	35.8	101	36.3	161	37.4	1134	32.1
Total	610	100.0	711	100.0	533	100.0	505	100.0	461	100.0	278	100.0	431	100.0	3529	100.0

χ2 = 794.228,P<0.001.

### Analysis of Endocrine Therapy

Of the 4211 patients, 1599 received ET, with a median age of 48.0 years (range, 21∼84 years) and a mean age of 48.8 (±10.37) years.

### Menstruation and Hormone Receptor Status

Among the 1599 patients who received ET, 999 (58.3%) were premenopausal and 600(41.7%) were menopausal. A total of 1416 (88.6%) patients were ER+ and/or PR+, 75 (4.7%) patients were ER-/PR-, and 108 (6.7%) patients had unknown receptor status (Table 3).

**Table 3:** Menstruation and hormone receptor status of patients receiving ET.

			menstrual status					
	total		pre-menopausal		post-menopausal			
HR type	N	%	n	%	n	%	?2	p
ER+/PR+	1069	66.9	693	69.4	376	62.7	29.26	<0.001
ER+/PR-	193	12.1	93	9.3	100	16.7		
ER-/PR+	154	9.6	107	10.7	47	7.8		
ER-/PR-	75	4.7	41	4.1	34	5.7		
Other types	13	0.8	11	1.1	2	0.3		
Not tested	77	4.8	42	4.2	35	5.8		
Unknown	18	1.1	12	1.2	6	1.0		
total	1599	100.0	999	100.0	600	100.0		

### ET Medications

**Figure 2:**
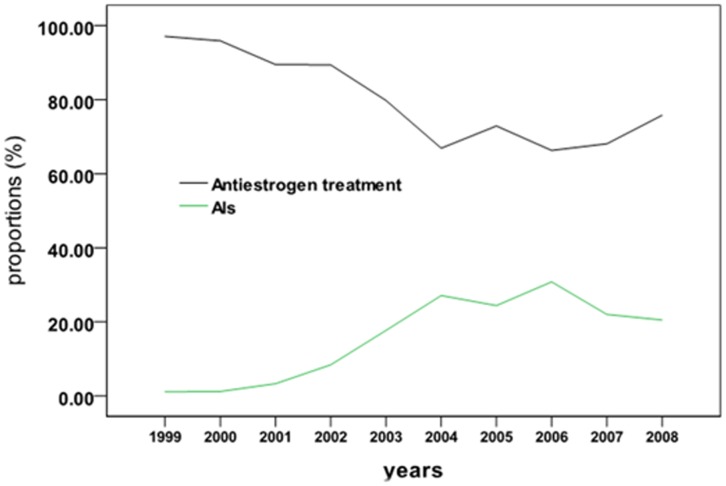
Ratios of the use of ET Drugs during 1990 and 2008.

Of the 1599 patients who received ET, 1598 (99.9%) received adjuvant ET, 13 (0.81%) patients received neoadjuvant ET, and 9 (0.56%) patients received palliative therapy after recurrence. Only 1 patient (0.1%) received palliative ET. Of the 1598 patients with adjuvant ET, 80.3 (1283/1598) received anti-estrogen therapy (tamoxifen or toremifene), but the proportion was trending down; the highest rate was 97.8% (177/181) and the lowest rate was 66.3%(114/172). Of the 1598 patients, 15.5% received AIs (anastrozole, letrozole, or exemestane) and the proportion was trending up (Figure 2).The highest proportion (89.7%[57/64]) of patients receiving anti-estrogen therapy resided in the northwest area while the highest proportion (23.4% [22/94]) of patients receiving AI therapy resided in the northeast (Tables 4 and 5).

**Table 4:** Medications used for ET during 1999 and 2008.

	Years										
	1999	2000	2001	2002	2003	2004	2005	2006	2007	2008	Total
method	n(%)	n(%)	n(%)	n(%)	n(%)	n(%)	n(%)	n(%)	n(%)	n(%)	n(%)
Tamoxifen	146(80.7)	140(81.9)	109(71.2)	87(65.9)	90(57.0)	72(47.7)	87(59.3)	86(50.0)	92(53.6)	84(52.2)	993(62.2)
Toremifene	31(17.1)	24(14.0)	28(18.3)	31(23.5)	36(22.8)	29(19.2)	20(13.6)	28(16.3)	25(14.5)	38(23.6)	290(18.1)
Anastrozole	0(0.0)	0(0.0)	0(0.0)	4(3.0)	7(4.4)	11(7.3)	9(6.1)	28(16.3)	15(8.7)	13(8.1)	87(5.4)
Letrozole	2(1.1)	2(1.2)	4(2.6)	6(4.5)	13(8.2)	18(11.9)	23(15.6)	15(8.7)	20(11.6)	6(3.7)	109(6.8)
Exemestane	0(0.0)	0(0.0)	1(0.7)	1(0.8)	8(5.1)	12(7.9)	4(2.7)	10(5.8)	3(1.7)	13(8.1)	52(3.3)
Others	0(0.0)	5(2.9)	8(5.2)	0(0.0)	0(0.0)	0(0.0)	0(0.0)	0(0.0)	1(0.6)	0(0.0)	14(0.9)
Unknown	2(1.1)	0(0.0)	3(2.0)	3(2.3)	4(2.5)	9(6.0)	4(2.7)	5(2.9)	16(9.3)	7(4.3)	53(3.3)
Total	181(100.0)	171(100.0)	153(100.0)	132(100.0)	158(100.0)	151(100.0)	147(100.0)	172(100.0)	172(100.0)	161(100.0)	1598(100.0)

χ2 = 317.4, p<0.001.

**Table 5:** Medications used for ET in different geographic regions from 1999 to 2008.

	Areas							
	Northern	Northeast	Central	South	Southeast	Northwest	Southwest	Total
medications	n(%)	n(%)	n(%)	n(%)	n(%)	n(%)	n(%)	n(%)
Tamoxifen	213(59.0)	69(73.4)	159(47.2)	211(58.3)	187(84.6)	57(89.1)	97(60.6)	993(62.1)
Toremifene	59(16.3)	0(0.0)	133(39.5)	89(24.7)	2(0.9)	0(0.0)	7(4.4)	290(18.1)
Anastrozole	45(12.5)	1(1.1)	14(4.2)	22(6.1)	2(0.9)	0(0.0)	3(1.9)	87(5.4)
Letrozole	22(6.1)	21(22.3)	15(4.5)	26(7.2)	16(7.2)	4(6.3)	5(3.1)	109(6.8)
Exemestane	17(4.7)	0(0.0)	16(4.7)	10(2.8)	9(4.1)	0(0.0)	0(0.0)	52(3.3)
Others	2(0.6)	2(2.1)	0(0.0)	2(0.6)	3(1.4)	1(1.6)	4(2.5)	14(0.9)
Unknown	3(0.8)	1(1.1)	0(0.0)	1(0.3)	2(0.9)	2(3.1)	44(27.5)	53(3.3)
Total	361(100.0)	94(100.0)	337(100.0)	361(100.0)	221(100.0)	64(100.0)	160(100.0)	1598(100.0)

χ2 = 650.86, p<0.001.

### Factors affecting ET in Breast Cancer patients with Positive Hormone Receptors

Of the 2395 patients with ER+ and/or PR+, 249 patients were excluded from logistic regression analysis no ET information was recorded in their medical records). Of the 2010 patients with complete ET information, 1384 (68.9%) received adjuvant ET whereas 626 (31.1%) did not receive adjuvant ET.

We performed logistic regression analysis on factors that might affect decision-making regarding the use of ET for breast cancer patients who were positive for hormone receptors. The results showed that cases undergoing ET were increased in the following groups of patients: a history of chemotherapy (P<0.05); a surgical history (P<0.05); residing in economically- developed areas (P<0.001); non-physical laborers (P<0.05). Age at the time of diagnosis, menstruation status, age of menopause, TNM stage, local invasion status, pathologic types, a history of radiotherapy, and education factor had no significant effect on decision-making regarding use of ET(P>0.05; Tables 6 and 7).

**Table 6:** Univariate logistic regression analysis for the use of ET.

		endocrine therapy					
		no (N = 626)		yes (N = 1384)			
Item		N	%	N	%	OR(95% CI)	P
Average age at diagnosis (years)						1.008(0.829–1.224)	0.94
	≤50	391	31.21	862	68.79		
	>50	235	31.04	522	68.96		
Menstrual status						1.077(0.884–1.313)	0.463
	Pre-menopausal	410	31.71	883	68.29		
	Menopausal	216	30.13	501	69.87		
Age of menopause						0.751(0.539–1.046)	0.09
	≤50	132	28.03	339	71.97		
	>50	84	34.15	162	65.85		
TNM staging						0.823(0.670–1.010)	0.063
	Early stage *	274	27.9	708	72.1		
	Advanced cancer	247	29.7	525	70.3		
Local infiltration						0.805(0.488–1.326)	0.394
	No	529	30.9	1183	69.10		
	Yes	25	35.71	45	64.29		
Pathologic type						1.005(0.616–1.640)	0.984
Carcinoma in situ (DCIS)^**^		24	30.4	55	69.6		
Non-specific infiltrative cancer ^***^		541	30.3	1246	69.7		
Specific type carcinoma ^****^		29	37.2	49	62.8		
Adjuvant chemotherapy						1.915(1.489–2.462)	<0.001
	No	132	44.1	167	55.9		
	Yes	490	29.2	1187	70.8		
Adjuvant radiotherapy						1.375(1.089–1.737)	0.008
	No	434	32.4	904	67.6		
	Yes	125	25.9	358	74.1		
Surgery						3.506(1.353–9.088)	0.01
	No	11	61.1	7	30.9		
	Yes	614	30.9	1370	69.7		
Geographic						2.052(1.688–2.496)	<0.001
Less developed areas ^a^		410	38.10	665	61.90		
Developed areas ^b^		216	23.10	719	76.90		
Education						1.077(0.818–1.417)	0.598
Junior high school or lower		160	27.7	418	72.3		
High school or higher ^c^		123	26.2	346	73.8		
Career						1.327(1.057–1.665)	0.015
Physical laborers		384	31.8	825	68.2		
Non-physical laborers^ d^		140	26.0	399	74.0		

Notes:

*Early breast cancers include stage 0, stage I, and stage II cancers;**Carcinomas *in situ* includes lobular and ductal carcinomas *in situ*, microinvasive carcinoma, and Paget's disease; ***Infiltrative non-specific cancers are invasive ductal and lobular carcinomas and mixed ductal carcinoma; ****Special carcinomas are tubular carcinoma, medullary carcinoma, and mucinous carcinoma.

a:Northeast, Central, Northwest, and Southwest areas; b:Northern, Southern, and Eastern areas; c: junior high school and below:primary school, junior high school, and illiteracy;senior high school and above: senior high school, junior college, and above degree;d: business staff, manual workers, housewives, soldiers, and others.

**Table 7:** Multivariate logistic regression analysis for the use of ET.

				95% CI for EXP(B)	
Item	S.E.	Sig.	Exp(B)	Lower	Upper
Chemotherapy	0.161	<0.001	8.774	6.405	12.018
Radiotherapy	0.213	0.387	1.202	0.792	1.826
Surgery	0.316	<0.001	26.971	14.517	50.109
Geographic	0.163	<0.001	1.996	1.449	2.750
Career	0.170	0.003	1.650	1.183	2.302

## Discussion

### The rate of hormone receptor testing increased year-after-year

Hormone receptors are biological markers which are most closely associated with the incidence, development, and prognosis of breast cancer and are needed to determine whether the breast cancer is invasive or not.

The rate of hormone receptor testing in our study was 83.8%, which was lower than the rate of 92.7% (72643/783431) reported by Li in the USA in 2002[31].The possible explanation for the discrepancy is that North America and Europe are the first areas in which clinical studies involving ET were conducted. In contrast, the use of hormone receptor testing was relatively late in China, and for a long time hormone receptor testing was not a routine post-surgical examination for breast cancer patients. Our study showed that the rate of hormone receptor testing in China increased from 72.2% in 1999 to 91.8% in 2008, and gradually approached the reported 92.7%.The common knowledge of hormone receptors increased in China and hormone receptor testing has been gradually popularized and developed. Nevertheless, the rate of hormone receptor testing for breast cancer needs to be further increased.

In the current study, significant geographic differences were detected in the rate of hormone receptor test, which was the lowest in the northwest area. The possible explanation for this observation is that there is a disparity in the economic level across China. The treatment decision and the choice of treatment regimen regarding ET for breast cancer are in fact affected by the economic level. Patients in less-developed regions have relatively low economic incomes and physicians and patients are unwilling to perform hormone receptor testing and ET for breast cancer.

### Analysis of the use of ET

A hormone receptor positive tumor is the primary indication for the use of ET for breast cancer patients. The effective rate of ET was 60%–70% for ER+/PR+ patients, 40% for ER+ or PR+ patients, and less than 10% for ER-/PR- patients. Our results provided evidence for the NIH and St.Gallen International Conference of Breast Cancer's suggestion that ER+ and/or PR+ breast cancer patients should receive post-operative adjuvant ET regardless of age, menstruation status, tumor size, and regional lymphatic metastasis status[25].Specifically, all hormone receptor-positive patients should undergo ET before and after surgery).

In the current study, 88.6% (1416/1599) of patients who received ET were positive for ER and/or PR, which is consistent with the previously reported rates (50%∼86%)[35]–[39]. Of the patients who received ET, 6.7% (108/1598) had unknown hormone receptor status, which was lower than the rate (15.8%) given by an Australian report [39]. The reason for the observed difference was that receptor testing was not popular in earlier years. Indeed, better treatment results can be achieved by the proper use of ET for 30% of patients who have unknown hormone receptor status[32]–[34]. To date, hormone receptor testing has become routine and patients with unknown receptor status has been decreasing in number in clinical practice.

### Patterns of ET

ET for breast cancer is used as a post-operative adjuvant therapy for early breast cancer[16]–[22] [40]; a neoadjuvant therapy for local advanced breast cancer[41]–[48]; or a palliative therapy for recurrent and metastatic breast cancer[49]. In the current study, most of the patients (99.9%) received adjuvant ET. Only 13 patients received neoadjuvant ET and 1 patient received palliative ET. The above-mentioned situations may be due to the fact that the medical records we retrospectively analyzed belonged to hospitalized patients and that follow-up information was not available. Moreover, the medical records of a number of patients were not available although a number of patients had indications for the use of ET and received ET in the outpatient department or other hospitals.

### Medications for ET

To date, anti-estrogens and AIs have been used as the common medications for ET [16], [50]–[52]. Our study showed that 80.3% (1283/1598) of the patients receiving ET were given anti-estrogens as the primary ET agents during 1999 and 200, which is consistent with Australian reports (73%∼83%) [39] [53] and higher than US and New Zealand reports (25%∼34.5%)[27] [54].The possible explanations for the discrepancy were as follows. First, most of the patients in our study suffered breast cancer before menopause, at an earlier age than the patients in Europe and the US. In the current study, 62.9% (2649/4211) of the patients had breast cancer before menopause. Of the patients undergoing ET, 58.3% (999/1599) were pre-menopausal patients. Second, AIs have recommended for adjuvant ET for menopausal patients in China since 2003. Third, anti-estrogens are generally used as the first-line ET drugs for patients with breast cancer because AIs are extremely expensive and are not listed on the medical insurance formulary.

Additionally, this study showed that the proportion of anti-estrogen drugs used for ET declined year after year from 1999 to 2008. In contrast, the proportion of AIs increased year by year from 2002 to 2006, which is consistent with the reports by Bowles[27] in the US. The above-mentioned status is partly due to the fact that AIs, which are representative of the ATAC, were not developed for clinical study and practice until 2001[55], [56]. However, AI use slightly declined in China after 2006, which was usually ascribed to physicians' better understanding of the indications and side effects of AIs and the availability of other medications for ET[27].

### Factors affecting the use of ET for Hormone Receptor-positive Breast Cancer Patients

China was stratified into seven geographic regions according to the traditional administrative district definition,such as North, North-East,Central, South, East, North-West, and South-West[28]. Since 1990, China' social economy reform and fast urbanization has impelled the rapid vicissitude of the urban and rural development. With that come the socioeconomic inequality, such as the prosperous coast in the Southern, Eastern, and Northern areas of China, and the relatively needier inland regions, such as Northeast, Central, Northwest, and Southwest areas.

By analyzing 2010 patients' complete information regarding endocrine therapy, we found that 68.9% (1384) received adjuvant ET while 31.1% (626) did not. The logistic regression analysis demonstrated that geography, occupation, and history of chemotherapy and surgery were dependent factors that affected the use of ET for hormone receptor-positive breast cancer patients in China. These results were in agreement with our national situations regarding the use of ET [40], [51], [57]. Many studies have shown that socioeconomic disparities including family income, educational attainment etc. were related to the diagnosis, methods of early medical treatment and prognosis of the disease [28], [57]–[59].In China, a large proportion of the costs of ET are paid by hospitalized patients and outpatients; thus, the family incomes of breast cancer patients play an important role in patients and physicians' decisions on whether ET should be used for breast cancer. ET was well-received in Northern, Southern, and Eastern China because these regions are more economically developed and people there have higher incomes, and then spend more on health care. Moreover, to achieve the expected efficacy, the patients with history of chemotherapy and surgery had better compliance with ET. They easily accepted and even insisted on ET [60]–[62].

In conclusion, ET for breast cancer in Chinese women is receiving increasing amounts of attention and is becoming the standard of care. Economic status, occupational factors, and history of chemotherapy and surgery were the key factors affecting the use of ET amongst breast cancer patients in China. People residing in developed areas, engaging in mental labour, having history of chemotherapy and surgery are susceptible to accept ET. Nevertheless, there were some limitations to our study. First, selection bias may exist in the catchment area of breast cancer patients in the selected hospitals as no less elite hospitals by comparison were selected from the same regions. Second, there was no comparison group to compare the risk factors for developing breast cancer. Third, data quality is dependent on the thoroughness of the documentation of medical history, treatment, and outcomes. Finally, unfortunately, the research subjects were only hospitalized patients, and followed with no effective follow-up. Such a situation resulted loss of data to a certain extent, not to mention the survival benefit.
